# Strong Impact of TGF-β1 Gene Polymorphisms on Breast Cancer Risk in Indian Women: A Case-Control and Population-Based Study

**DOI:** 10.1371/journal.pone.0075979

**Published:** 2013-10-17

**Authors:** Singh Pooja, Amirtharaj Francis, Singh Rajender, Rakesh Tamang, Raja Rajkumar, Karan Singh Saini, Kaling Megu, Madhu Mati Goel, Daminani Surekha, Digumarthi Raghunatha Rao, Lakshmi Rao, Lingadakai Ramachandra, Sandeep Kumar, Surender Kumar, Satti Vishnupriya, Kapaettu Satyamoorthy, Mahendra Pal Singh Negi, Kumarasamy Thangaraj, Rituraj Konwar

**Affiliations:** 1 Endocrinology Division, CSIR-Central Drug Research Institute, Lucknow, India; 2 Department of Pathology, King George's Medical University, Lucknow, India; 3 CSIR-Centre for Cellular and Molecular Biology, Hyderabad, India; 4 Department of Genetics, Osmania University, Hyderabad, India; 5 Department of Surgery, King George's Medical University, Lucknow, India; 6 Nizam's Institute of Medical Sciences, Hyderabad, India; 7 Department of Pathology, Kasturba Medical College, Manipal University, Manipal, India; 8 Department of Surgery, Kasturba Medical College, Manipal University, Manipal, India; 9 All India Institute of Medical Sciences, Bhopal, India; 10 Manipal Life Sciences Center, Manipal University, Manipal, India; 11 Biometric and Statistics Division, CSIR-Central Drug Research Institute, Lucknow, India; Health Canada and University of Ottawa, Canada

## Abstract

**Introduction:**

TGF-β1 is a multi-functional cytokine that plays an important role in breast carcinogenesis. Critical role of TGF-β1 signaling in breast cancer progression is well documented. Some TGF-β1 polymorphisms influence its expression; however, their impact on breast cancer risk is not clear.

**Methods:**

We analyzed 1222 samples in a candidate gene-based genetic association study on two distantly located and ethnically divergent case-control groups of Indian women, followed by a population-based genetic epidemiology study analyzing these polymorphisms in other Indian populations. The c.29C>T (Pro10Leu, rs1982073 or rs1800470) and c.74G>C (Arg25Pro, rs1800471) polymorphisms in the TGF-β1 gene were analyzed using direct DNA sequencing, and peripheral level of TGF-β1 were measured by ELISA.

**Results:**

c.29C>T substitution increased breast cancer risk, irrespective of ethnicity and menopausal status. On the other hand, c.74G>C substitution reduced breast cancer risk significantly in the north Indian group (p = 0.0005) and only in the pre-menopausal women. The protective effect of c.74G>C polymorphism may be ethnicity-specific, as no association was seen in south Indian group. The polymorphic status of c.29C>T was comparable among Indo-Europeans, Dravidians, and Tibeto-Burmans. Interestingly, we found that Tibeto-Burmans lack polymorphism at c.74G>C locus as true for the Chinese populations. However, the Brahmins of Nepal (Indo-Europeans) showed polymorphism in 2.08% of alleles. Mean TGF-β1 was significantly elevated in patients in comparison to controls (p<0.001).

**Conclusion:**

c.29C>T and c.74G>C polymorphisms in the TGF-β1 gene significantly affect breast cancer risk, which correlates with elevated TGF-β1 level in the patients. The c.29C>T locus is polymorphic across ethnically different populations, but c.74G>C locus is monomorphic in Tibeto-Burmans and polymorphic in other Indian populations.

## Introduction

Transforming growth factor beta (TGF-β) signaling is one of the most commonly altered cellular pathways in human cancers [1]–[4]. TGF-β1 is a multi-functional cytokine that plays an important role in breast carcinogenesis [5]. TGF-β1 is a potent inhibitor of proliferation of epithelial, endothelial and hematopoietic cells, and it acts as a tumor suppressor. TGF-β1 has dual role in carcinogenesis with tumor suppressive effects in epithelial cells, but tumor invasion and metastasis promoting effects during later stages of carcinoma progression [6]–[8]. Specific pathways are involved in the conversion of pro- and anti-tumor roles of TGF-β1 [9]. A majority of breast cancers secrete elevated TGF-β1 in tumor micro-environment associated with either malignant epithelial cells, stromal cells or both [10]. Increased immuno-reactivity for TGF-β protein correlates with poor prognosis and increased lymph node involvement [11], and elevated TGF-β associate with tamoxifen resistance [12]. The role of TGF-β has been widely recognized in cancer stem cells [13], [14] and TGF-β signaling in breast cancer has been extensively reviewed [15]. Eventually, TGF-β is thought of as a potential target for management of cancer [16]–[18] and inhibition of TGF-β has been tried for treating cancer, but without significant success till now [19]–[28].

TGF β are known as low penetrance genes in cancer [29]. There are three isoforms of TGF-β (TGF-β1, TGF-β2, and TGF-β3), of which TGF- β1 is most widely expressed [30]. TGF-β1 gene is located on chromosome 19q13.1 (OMIM 190180) [31]. So far, several polymorphisms in the TGF-β1 gene have been reported and found to affect TGF-β1 protein expression [32]. Relationship between TGF-β1 polymorphisms and breast cancer has been studied in several populations and is subject of further research interest due to lack of consensus in the data [33]–[41]. One of the most commonly studied polymorphisms in the TGF-β1 gene is c.29C>T substitution (rs1800470), resulting in proline (CCG) to leucine (CTG) change at codon 10 (Pro10Leu) of the protein (29). Another substitution, c.74 G>C (rs1800471), resulting in replacement of arginine (CGG) with proline (CCG) at codon 25 (Arg25Pro) of the protein, has been relatively less studied [42]. c.29C>T substitution results in increased secretion of cytokine [43], making it a strong candidate for analysis in breast cancer. These polymorphisms have not been widely analyzed in Indian populations, except the analysis of c.29C>T polymorphism in some Indian populations [44]–[46].

We conducted the present case-control study on a fairly large sample size to; 1) investigate the association between TGF-β1 polymorphisms (c.29C>T and c.74G>C) and breast cancer risk in India, 2) evaluate variation of the association across ethnically different populations, 3) compare genotype frequencies of these polymorphisms between Dravidian, Indo-European and Tibeto-Burman populations of India, and 4) compare TGF-β1 genotypes with other Asian populations from medico-evolutionary point of view.

## Materials and Methods

### Study subjects

#### Ethics statement

This case-control study was carried out with the approval of the Ethics Committee of the King George's Medical University, Lucknow. The subject recruitment and sample collection were done only after obtaining written informed consent of the participants.

The north Indian group, consisting of 113 patients and 113 control samples, was recruited from the Department of Surgery, King George's Medical University, Lucknow. The South Indian group, consisting of 352 patients and 126 control samples, was recruited from the Rai Memorial Hospital, Chennai, Nizam's Institute of Medical Sciences, Hyderabad, and Kasturba Medical College, Manipal University, Manipal. Women with histopathologically confirmed diagnosis of breast cancer were recruited as cases. Women visiting the clinic for problems other than breast cancer were recruited as controls after proper clinical investigation and/or a mammogram confirming no evidence of breast cancer. Women with any breast disorder or other systemic inflammatory disease were excluded from the control group. General health history of the cases and controls was collected with an appropriately designed proforma. A detailed description of the general and clinical characteristics of the patients is provided in Table 1.

**Table 1:** Participant characteristics.

	North Indian		South Indian	
Variables	Cases (N = 113)	Controls (N = 113)	Cases (N = 352)	Controls (N = 126)
Age (mean±SD)	45.42±15.56	41±17.30	49.52±13.32	49.21±11.71
BMI (Kg/m^2^)	21.64±6.21	23.19±5.14	20.18±5.70	21.19±5.71
Age at menarche (years, mean ± SD)	14.15±1.87	14.12±2.10	13.59±1.87	13.51±1.88
**Age at diagnosis for cases or at interview for controls**				
≤30 years	18 (15.93%)	12 (10.62%)	7 (1.99%)	8 (6.35%)
31–45 years	36 (31.86%)	48 (42.48%)	109 (30.97%)	44 (34.92%)
46–60 years	38 (33.63%)	33 (29.20%)	155 (44.03%)	52 (41.27%)
61–75 years	15 (13.27%)	14 (12.39%)	75 (21.31%)	19 (15.08%)
76–90 years	6 (5.31%)	6 (5.31%)	6 (1.71%)	3 (2.38%)
**Family history**				
Positive	13 (11.50%)	0 (0%)	40 (11.36%)	0 (0%)
Negative	100 (88.50%)	113 (100%)	312 (88.64%)	126 (100%)
**Tobacco chewing/smoking habit**				
Yes	12 (10.62%)	5 (4.42%)	16 (4.55%)	4 (3.17%)
No	101 (89.38%)	108 (95.58%)	336 (95.55%)	122 (96.83%)

Three (Dravidian, Indo-European, and Tibeto-Burman) out of four major linguistic groups, inhabiting the Indian mainland, have been included in this study. After analyzing Indo-European case-control group from northern India and Dravidian case-control group from southern India, we extended the analysis to the Tibeto-Burman populations from north-eastern India. Striking differences in the allele frequency between Indian and East-Asian (Chinese) populations [47], particularly at the c.74G>C locus [48], encouraged us to genotype both the SNPs in Tibeto-Burman populations, in order to further explore the medico-evolutionary significance of TGF- β1 polymorphisms. Tibeto-Burmans in India have close genetic affinities with East Asian populations [49]. We recruited a total of 508 Tibeto-Burmans from north-eastern regions of India, Nepal, and those residing in other states of India. Samples were collected from Khasi of Meghalaya, Ao-Naga, Naga Sema, and Chakhesang Naga of Nagaland, Nyshi of Arunachal Pradesh, Mizo of Mizoram, Poumai Naga of Manipur, Sherpa and Subba of Darjeeling (West Bengal), and Tibeto-Burmans residing in Mysore (Karnataka). Since both Indo-European and Tibeto-Burman populations inhabit Nepal, we recruited Nepali Brahmins (Indo-European) and Magar community (Tibeto-Burman) people to compare the genotype frequency with other populations of South-East Asia.

### Genotyping

Isolation of DNA for genotyping was carried out as described in our earlier report [50]. The target TGF-β1 fragment was amplified using primers, GAGGCCCTCCTACCTTTTG (F) and GCAGCTTGGACAGGATCT (R), and PCR products were analyzed on 2% agarose gel stained with ethidium bromide. The amplified products were analyzed by direct DNA sequencing using big dye chain terminator cycle sequencing kit (ABI) on a 3730 DNA analyzer (Applied Biosystems).

### Statistical analysis

Genotype data for control population were subjected to test for fitness in the Hardy Weinberg equilibrium. Statistical computational software available at http://ihg.gsf.de/cgi-bin/hw/hwa1.pl was employed for this purpose. The frequencies of the two alleles at the polymorphic sites were compared between cases and controls to find the risk allele. Genotype data were compared using 2×3 contingency table of Chi Square test or Fisher's exact test using statistical computational tools available at http://www.vassarstats.net. Fisher exact P values were calculated using 2×2 or 2×3 contingency tables, but wherever the software could not calculate Fisher exact values due to large sample size, Chi Square P value was used. Peripheral values of TGF- β1 were compared between cases and controls using non-parametric Mann-Whitney U-test. Age dependent multivariate Cox regression analysis was used to assess the genotype associated risk factors of breast cancer, considering genotypes as a risk event and socio-demographic factors as other variables (confounder covariates). Two sided P-values of less than 0.05 were considered significant for statistical inference.

## Results

### Subject characteristics

We did not find any statistically significant difference in general characteristics between cases and controls (Table 1). However, slightly more number of breast cancer patients in the north Indian group fall in the younger age group (15.93% versus 1.99%, Table 1). More than 88% of breast cancer patients in both north Indian and south Indian groups were sporadic. The incidence of familial breast cancer in our subject population was quite high at about 11% frequency, which is lower than reported in other populations. Apparently, there was no correlation between tobacco chewing or smoking and the incidence of breast cancer in the study population.

### TGF-β c.29C>T (codon 10) polymorphism

Genotype data were in Hardy Weinberg equilibrium for both north Indian (F = 0.0186, Exact P = 1.0) and south Indian groups (F = 0.0648, Exact P = 0.586). Analysis of the pooled data for all breast cancer patients versus controls showed that C>T substitution increased breast cancer risk (p = 0.00007 for allele comparison and 0.000003 for genotype comparison) (Table 2). Group-wise analysis showed that C>T substitution at codon 10 increased breast cancer risk both in north Indian (p = 0.0012 for allele comparison and 0.0037 for genotype comparison) and south Indian groups (p = 0.0413 for allele comparison and 0.0004 for genotype comparison) (Table 3). Sub-group analysis showed that C>T substitution increases breast cancer risk in the north Indian group, irrespective of menopause status (Table 4). However, in south Indians, though the association was significant in the post-menopausal women, it is only marginally significant in pre-menopausal women (Table 4).

**Table 2:** Pooled data comparison for all cases versus controls.

All subjects (Codon 10)					
Alleles					
	C	T	Comparison	OR (95% CI)	
Cases	335 (36.10)	593 (63.90)	Fisher exact P = 0.00007*	1.58 (1.26–1.98)	
Controls	225 (47.27)	251 (52.73)			
**Genotype**					
	CC	CT	TT	Comparison	OR (95% CI)
Cases	85 (18.32)	165 (35.56)	214 (46.12)	Chi Square P = 0.000003*	—
Controls	51 (21.43)	123 (51.68)	64 (26.89)		

All subjects (Codon 25)					
Alleles					
	G	C	Comparison	OR (95% CI)	
Cases	881 (94.73)	49 (5.27)	Chi Square P = 0.0636	0.66 (0.42–1.02)	
Controls	439 (92.23)	37 (7.77)			
**Genotype**					
	GG	GC	CC	Comparison	OR (95% CI)
Cases	419 (90.11)	43 (9.25)	3 (0.64)	Chi Square P = 0.1653	—
Controls	203 (85.29)	33 (13.87)	2 (0.84)		

Allele and genotype frequency is followed by percent values in parenthesis.

* Indicates statistical significant value at 95% level of confidence.

**Table 3:** +29 C>T polymorphism allele and genotype data comparison between cases and controls.

North Indian group					
Alleles					
	C	T	Comparison	OR (95% CI)	
Cases	80 (35.71)	144 (64.28)	Fisher exact P = 0.0012*	1.89(1.30–2.77)	
Controls	115 (51.33)	109 (48.66)			
**Genotype**					
	CC	CT	TT	Comparison	OR (95% CI)
Cases	16 (14.28)	48 (42.85)	48(42.85)	Fisher exact P = 0.0037*	—
Controls	29 (25.89)	57 (50.89)	26(23.21)		

South Indian group					
Alleles					
	C	T	Comparison	OR (95% CI)	
Cases	255 (36.22)	449 (63.77)	Fisher exact P = 0.0413*	1.36 (1.02–1.83)	
Controls	110 (43.65)	142 (56.34)			
**Genotype**					
	CC	CT	TT	Comparison	OR (95% CI)
Cases	69(19.60)	117(33.23)	166(47.15)	Chi Square P = 0.0004*	—
Controls	22(17.46)	66(52.38)	38(30.15)		

Allele and genotype frequency is followed by percent values in parenthesis.

* Indicates statistical significant value at 95% level of confidence.

**Table 4:** Comparison of +29 C>T genotype data between case groups as per menopause and the controls.

North Indian group					
	Controls (112)	Pre-menopausal patients (65)	P value, OR (95% CI)	Post-menopausal patients (47)	P value, OR (95%CI)
**Alleles**					
**C**	115(51.33)	46(35.38)	Fisher exact P = 0.004*, 1.93 (1.23–3.00)	34(36.17)	Fisher exact P = 0.014*,1.86 (1.13–3.06)
**T**	109(48.66)	84(64.61)		60(63.82)	
**Genotypes**					
**C/C**	29(25.89)	8(12.30)	Fisher exact P = 0.015*	8(17.02)	Fisher exact P = 0.029*
**C/T**	57(50.89)	30(46.15)		18(38.29)	
**T/T**	26(23.21)	27(41.53)		21(44.68)	

South Indian Group					
	Controls (126)	Pre-menopausal patients (128)	P value, OR (95%CI)	Post-menopausal patients (224)	P value, OR (95%CI)
**Alleles**					
**C**	110(43.65)	100(39.06)	Fisher exact P = 0.321,1.21 (0.85–1.72)	155(34.59)	Fisher exact P = 0.018*,1.46 (1.07–2.01)
**T**	142(56.34)	156(60.93)		293(65.40)	
**Genotypes**					
**CC**	22(17.46)	26(20.47)	Fisher exact P = 0.043*	43(19.11)	Chi Square P = 0.0002*
**CT**	66(52.38)	47(37.00)		70(31.11)	
**TT**	38(30.15)	54(42.51)		112(49.77)	

Allele and genotype frequency is followed by percent values in parenthesis.

* Indicates statistical significant value at 95% level of confidence.

### TGF-β c.74G>C (codon 25) polymorphism

Genotype data for this polymorphism were found to be in Hardy Weinberg equilibrium for both north Indian (F = 0.031, Exact P = 0.656) and south Indian groups (F = 0.0413, Exact P = 1.0). Analysis of the pooled data for both the study groups showed that codon 25 polymorphism was not associated with breast cancer risk (p = 0.063 for allele comparison and 0.165 for genotype comparison) (Table 2). In group-wise analysis, a significant association was observed in the north Indian group (p = 0.0016 for allele comparison and 0.0018 for genotype comparison) (Table 5) such that the substitution was protective against breast cancer. However, the polymorphism showed no association in case of south Indian group (p = 0.327 for allele comparison and 0.554 for genotype comparison) (Table 5). In sub-group analysis on the basis of menopause status, the difference was significant only in the pre-menopausal group of north Indian women (p = 0.011 for allele comparison and p = 0.005 for genotype comparison) (Table 6). However, in post-menopausal group, no difference between cases and controls at genotype level was seen (p = 0.104). The frequencies of the two alleles and the genotypes at this site were comparable between south Indian cases and controls (Table 5), and the protective effect as seen in the north Indian group, was not evident in the South Indian group (Tables 5 and 6).

**Table 5:** Comparison of +74 G>C genotype data between cases and controls.

North Indian group					
Alleles					
	G	C	Comparison	OR (95% CI)	
Cases	217 (96.017)	9 (3.98)	Fisher exact P = 0.0016*	0.30 (0.14–0.66)	
Controls	197 (87.94)	27 (12.053)			
**Genotype**					
	GG	GC	CC	Comparison	OR (95% CI)
Cases	105(92.92)	7(6.19)	1 (0.88)	Fisher exact P = 0.0018*	—
Controls	87(77.67)	23(20.53)	2 (1.78)		

South Indian group					
Alleles					
	G	C	Comparison	OR (95% CI)	
Cases	664 (94.31)	40 (5.68)	Fisher exact P = 0.327	1.45 (0.72–2.96)	
Controls	242 (96.03)	10 (3.96)			
**Genotype**					
	GG	CG	CC	Comparison	OR (95% CI)
Cases	314(89.204)	36(10.227)	2(0.568)	Chi Square P = 0.554	—
Controls	116(92.06)	10(7.936)	0(0)		

Allele and genotype frequency is followed by percent values in parenthesis.

* Indicates statistical significant value at 95% level of confidence.

**Table 6:** Comparison of +74 G>C genotype data between case groups as per menopause and the controls.

North Indian group					
	Controls (112)	Pre-menopausal patients (65)	P value, OR (95% CI)	Post-menopausal patients (48)	P value, OR (95%CI)
**Alleles**					
**G**	197(87.94)	125(96.15)	Fisher exact P = 0.011*, 0.29 (0.11–0.78)	92(95.83)	Fisher exact P = 0.037*,0.32 (0.11–0.93)
**C**	27(12.05)	5(3.84)		4(4.16)	
**Genotypes**					
**GG**	87(77.67)	61(93.84)	Fisher exact P = 0.005*	44(91.66)	Fisher exact P = 0.104
**GC**	23(20.53)	3(4.61)		4(8.33)	
**CC**	2(1.78)	1(1.53)		0(0)	

South Indian Group					
	Controls (126)	Pre-menopausal patients (127)	P value, OR (95%CI)	Post-menopausal patients (225)	P value, OR (95%CI)
**Alleles**					
**G**	136(93.15)	236(92.91)	Fisher exact P = 1.000, 1.03 (0.46–2.31)	428(95.11)	Fisher exact P = 0.397,0.69 (0.32–1.51)
**C**	10(06.84)	18(07.62)		22(4.88)	
**Genotypes**					
**GG**	116(92.06)	111(87.40)	Fisher exact P = 0.264	203(90.22)	Chi square P = 0.847
**GC**	10(7.93)	14(11.02)		22(9.77)	
**CC**	0(00.00)	2(1.57)		0(00.00)	

Allele and genotype frequency is followed by percent values in parenthesis.

* Indicates statistical significant value at 95% level of confidence.

**Figure 1:**
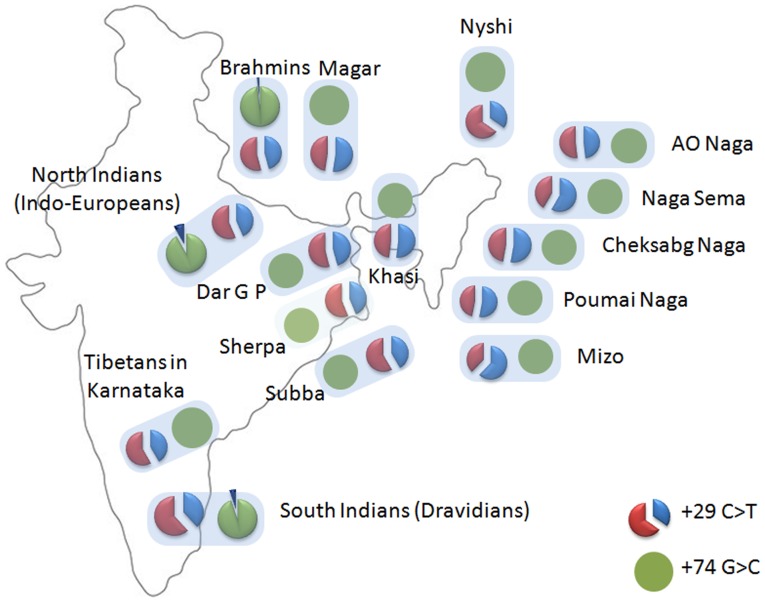
Distribution of the two polymorphisms in Indian populations.

The polymorphic status of +29C>T was comparable among the Indo-European (North), Dravidian (South), and the Tibeto-Burman (North-East) Indian populations (Figure 1). Interestingly, +74G>C substitution was observed in the Indo-European and Dravidian populations at a frequency of 5–8%, but was completely absent in Tibeto-Burmans. Tibeto-Burmans invariably possessed ‘GG’ genotype at +74 G>C locus. The Magar group (Tibeto-Burman) of Nepal also did not exhibit any polymorphism at this locus. However, the Brahmins of Nepal (Indo-European) showed polymorphism frequency comparable to other Indo-European populations. It is clear that the polymorphism at c.29C>T locus is very common and widespread. On the other hand, c.74G>C locus is polymorphic in the Dravidian and Indo-European populations, but completely monomorphic in the Tibeto-Burman populations of India, irrespective of the location and caste status.

### Serum level of TGF-β1 in breast cancer patients and control subjects

**Figure 2:**
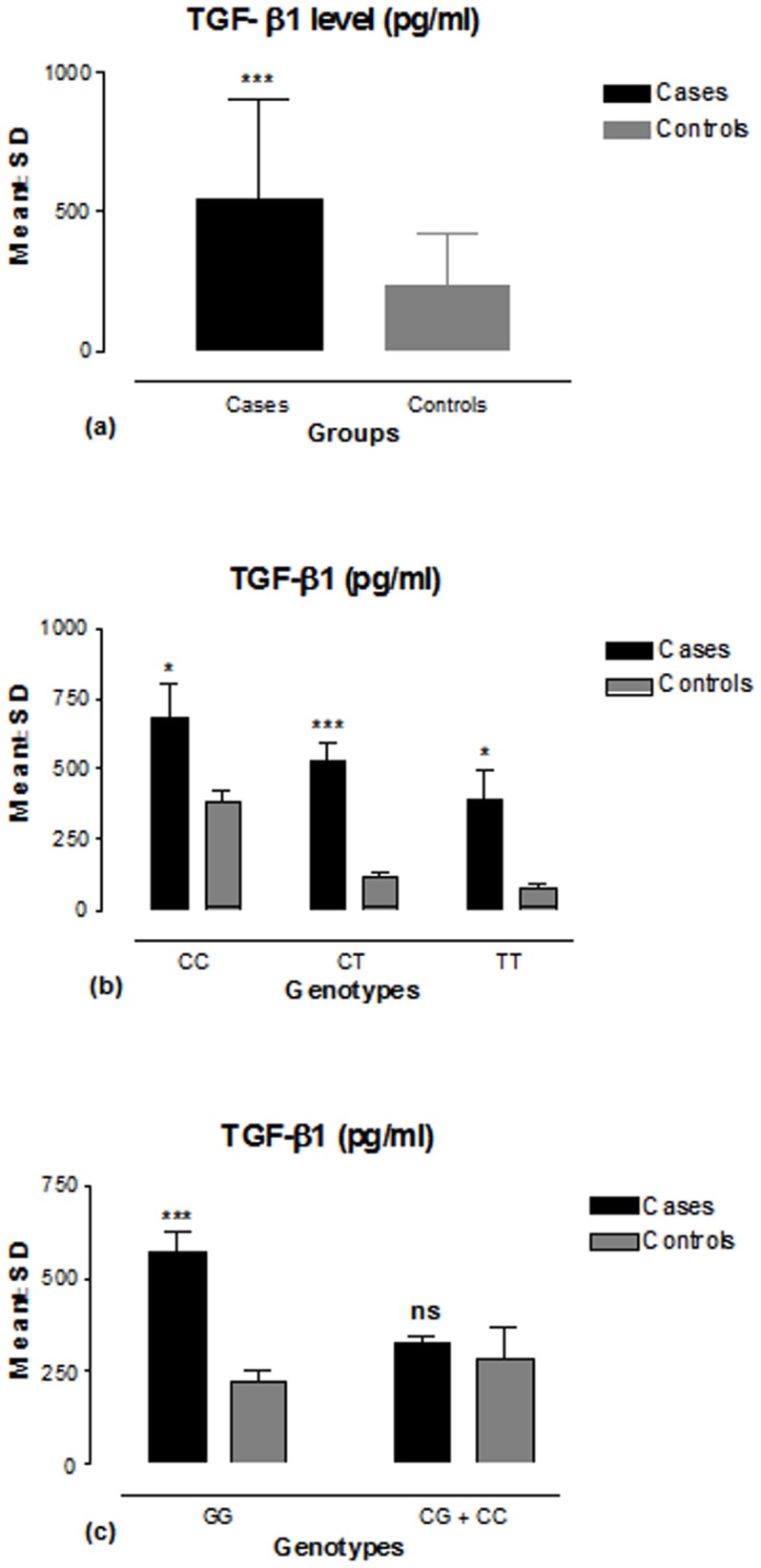
Peripheral level of TGF-β1 (a) in cases and controls, (b) according to genotypes at c.29C>T and (c) c.74G>C locus. Significance level: non-significant - p>0.05, *- p<0.05, ***- p<0.001.

We also measured the serum level of TGF-β1 in a subset of cases and controls of the North Indian group (Figure 2). Peripheral mean TGF-β1 level in the cases was significantly (U = 324.00, p<0.001) higher in comparison to the controls (Figure 2a). Further, the mean TGF-β1 level in cases across all three genotypes (CC: U = 72.00, p = 0.028; CT: U = 3.00, p<0.001; and TT: U = 11.00, p = 0.042) at +29 C>T polymorphism was also found to be significantly higher as compared to the controls (Figure 2b). In contrast, the GG genotype at +74G>C polymorphism showed significantly (U = 212.00, p<0.001) higher mean TGF-β1 level in cases as compared to controls, but TGF-β1 level in case of CG+CC genotypes did not differ significantly between the two groups (U = 9.00, p = 0.630, Figure 2c).

### Association of TGF-β polymorphism with covariates

To determine the predictors (covariates) of breast cancer risk, genotype data for both the polymorphisms (c.29C>T and c.74G>C) of North Indians were further analyzed by multivariate Cox regression (Table 7). None of the investigated covariates showed significant association with genotypes associated breast cancer risk, except the menopausal status. The menopausal status in both the polymorphisms showed significant (p<0.001) association with breast cancer risk.

**Table 7:** Age dependent associations of genotypes with confounding covariates in north Indian population by Cox regression analysis.

Predictors	+29C>T		+74G>C	
	OR (95% CI)	P value	OR (95% CI)	P value
BMI	1.15 (0.67–1.97)	0.608	1.15 (0.68–1.97)	0.602
Diet	1.07 (0.62–1.85)	0.814	1.07 (0.63–1.83)	0.802
Relation	1.35 (0.55–3.30)	0.508	1.36 (0.56–3.30)	0.499
Family type	1.00 (0.46–2.15)	0.998	1.00 (0.46–2.14)	0.995
Personal habits	0.95 (0.43–2.13)	0.905	0.95 (0.43–2.12)	0.901
Age at menarche	0.80 (0.44–1.43)	0.444	0.79 (0.44–1.42)	0.435
Age at 1^st^ full term pregnancy	0.83 (0.50–1.38)	0.469	0.83 (0.50–1.37)	0.460
Menopausal status	3.38 (1.77–6.46)	<0.001*	3.43 (1.80–6.52)	<0.001*

The odds are of BMI “>23 kg/m^2^, Diet “Non vegetarian”, Religion “Hindu”, Family history “Yes”, Personal habits “Yes”, Age at menarche “>14 yrs), Age at 1^st^ full term pregnancy “>19 yrs” and Menopausal status “Yes” against BMI “≤23 kg/m^2^, Diet “Vegetarian”, Religion “Muslim”, Family history “No”, Personal habits “No”, Age at menarche “≤14 yrs), Age at 1^st^ full term pregnancy “≤19 yrs” and Menopausal status “No”.

* Statistically significant (p<0.001).

## Discussion

We observed that the c.29C>T substitution at codon 10 of the TGF-β1 gene significantly increases the risk of breast cancer in Indian populations. The patients exhibited a far higher frequency of the substitution in comparison to the controls. We found that the allele frequency at this locus in Indian populations is comparable to other populations across the globe (refer NCBI database). In sub-group analysis, we found this substitution to increase breast cancer risk irrespective of ethnicity, as both North- and South-Indian women having substitution were at an increased risk of breast cancer. Comparison of the pre-menopausal and post-menopausal cases with all controls suggested that c.29C>T substitution increases breast cancer risk irrespective of the menopausal status. Three other studies from India have analyzed c.29C>T locus in breast cancer [44]–[46]. Two of them reported no association of this polymorphism with breast cancer risk [44], [45]; however, Joshi et al. (2011) reported that TGF-β1 *29C was protective against breast cancer and suggested this to be a plausible reason behind relatively lower incidence of breast cancer in western Indian women in comparison to white women [46]. The allele and genotype frequencies in our study were comparable to those in Joshi et al (2011), and the data support that *29C is a protective allele and *29T a risk allele. Nevertheless, it is worth noting that our inference is in contrast to two other studies from India [44], [45].

Published data on c.29C>T polymorphism in breast cancer lack consensus. As a result, five meta-analyses have been conducted on this polymorphism. Interestingly, all five meta-analyses were published in the same year [29], [42], [51], [52], [53]. Two of these meta-analysis stated no association between c.29C>T polymorphism and breast cancer [29], [51], while the two others stated no overall association between this substitution and breast cancer risk, but an increased risk of breast cancer with 10P allele in Caucasians [42], [52], and yet another meta-analysis stated significant association of 10P in overall analysis as well as in the Caucasian group [53]. Contrary to the observations of all these meta-analyses, particularly the latter three, we found the alternate allele (‘T’ or ‘leucine’) to be a risk factor for breast cancer. Our results have infused further curiosity regarding the association of this polymorphism with breast cancer.

We observed that c.74G>C substitution was significantly protective against breast cancer in the north Indian population only. North Indian patient population exhibited a higher frequency of the substitution in comparison to the controls. Sub-grouping of North Indian cases according to the menopausal status revealed significant protective effect of this substitution in case of pre-menopausal women only. A clear ethnicity based impact on breast cancer risk of the genotypes at c.74G>C site was evident, as the protective effect of ‘CC’ genotype was not seen in the South Indian group. This polymorphism has been relatively less studied in comparison to c.29C>T substitution. Only one study on breast cancer from India has analyzed this polymorphism, finding no significant difference between cases and controls [44]; however, this study had severely low statistical power due to the use of a very small sample size for inference. Two other Indian studies on TGF- β1 polymorphisms in breast cancer did not analyze this polymorphism [45], [46]. We are the first to genotype this polymorphism in a significantly large sample size and report protective effect of the substitution. Our analysis on Tibeto-Burman populations of India found no variation at this locus. This observation is interesting, but not surprising, as one of our earlier studies showed complete absence of a 25 bp deletion polymorphism in the *MyBPC3* gene (causing various forms of cardiomyopathy) in these populations despite its presence in almost all other Indian populations at about 4% frequency [47]. Shanghai breast cancer study also found no incidence of sequence variation at c.74G>C locus after analysis on a cohort of 1111 Chinese patients [48]. Most other populations across the world exhibit small frequency of ‘C’ allele, showing widespread existence of this polymorphism (refer NCBI database).

Highly polymorphic status of the c.29C>T locus among Indian and North-Eastern Indian populations shows widespread existence of this polymorphism. Monomorphism at the c.74G>C locus unveils important medical and evolutionary significance associated with this locus. The absence of the protective allele (C) may suggest relatively higher risk of breast cancer in the Tibeto-Burmans in comparison to the Dravidians and Indo-Europeans. Similarly, the absence of ‘C’ allele in the Chinese populations may indicate increased breast cancer risk in comparison to the Indian populations. This notion is supported by a higher incidence of breast cancer in the Chinese populations in comparison to the Indian populations (Dravidian) as reported in an epidemiological study comparing breast cancer incidence over a period of three decades [54]. From evolutionary point of view, our data further supports the proposal that the people of north-eastern region of Indian are genetically closer to Chinese/East Asian populations [49].

We observed that TGF-β1 level in the breast cancer patients was significantly elevated as compared to the control group. The elevated TGF-β1 level could be due to a higher frequency of the risk genotypes in the cases. Further analysis on the basis of genotypes suggested that TGF-β1 level of cases in comparison to control was significantly higher in all the genotypes of c.29C>T locus, while in case of c.74G>C locus, it was only significant in absence of “C” allele. Intra-tumoral expression of TGF-β1 has been found to be significantly higher in invasive breast cancer patients [55]. It is well documented that TGF-β1 polymorphic variants are functionally associated with the level of TGF-β1 expression [40], [56]–[57]. Therefore, it is plausible that TGF-β1 polymorphisms affect breast cancer risk by modulating the level of TGF-β1 expression. In multistage progression of tumors, TGF-β exerts growth inhibitory effects in the initial phase; however, growth-inhibitory effects are abolished and malignant tumor promoting action of TGF-β is activated in the later stages [58]. Significant correlation of TGF-β1 allelic variants with elevated TGF-β1 level suggests their critical role in deciding cancer initiation and progression. Nevertheless, a direct correlation between allelic variants, the level of expression, and cancer risk or progression is difficult to derive since the level of TGF- β1 expression and its pro- and anti-apoptotic effects may differ at different stages of cancer progression. A stage specific analysis of the TGF- β1 expression level and haplotype analysis of all the polymorphisms of this locus could help further understand the breast cancer risk associated with TGF-β1 variations. We feel that availability of further details such as ER and HER2 status, treatment outcome, recurrence rate, and drug resistance data could have helped undertake further detailed investigations, which could not be undertaken due to unavailability of such data.

In conclusion, c.29C>T substitution increases breast cancer risk irrespective of ethnicity and menopausal status. This polymorphism is quite common across the world. c.74G>C polymorphism, on the other hand, showed ethnic variations such that the substitution decreased breast cancer risk in the north Indian populations, but not in their south Indian counterparts. This could be due to a significant impact of other co-occurring genetic variations affecting the risk due to this polymorphism. In other words, the genetic background perhaps becomes more influential in case of c.74G>C polymorphism. The c.74G>C locus is polymorphic across the world with moderate frequency of ‘CC’ genotype, except in case of the North-East Indians, Nepalese, and Chinese populations. Monomorphism at this locus may suggest increased breast cancer risk in these populations in comparison to other ethnic groups. The increased level of TGF- β1 in the patients in comparison to the controls could suggest the possible mechanism of the effect of TGF-β1 polymorphisms on breast cancer. However, further *in vitro* studies are required in order to decipher the mechanism of increased cancer risk in the carriers of certain TGF-β1 genotypes. Significant impact of c.74G>C polymorphism on breast cancer risk encourages more studies on this polymorphism. In addition to identifying genetic risk factors for breast cancer, our study has revealed striking differences in the genetic variations between different ethnic groups, which could have important implications on human health.
